# Implementing a Doctor-to-Doctor Telemedicine Network to Strengthen Rural Obstetric Care in Japan: Feasibility, Clinical Utility, and Educational Impact

**DOI:** 10.7759/cureus.102110

**Published:** 2026-01-22

**Authors:** Yoshitsugu Chigusa, Hikaru Imatake, Maya Komatsu, Yoshikazu Ikeda, Haruta Mogami, Masaki Mandai

**Affiliations:** 1 Gynecology and Obstetrics, Graduate School of Medicine, Kyoto University, Kyoto, JPN; 2 Obstetrics and Gynecology, Yasaka Hospital, Kyotango, JPN

**Keywords:** doctor-to-doctor consultation, healthcare disparities, medical education, perinatal care, rural healthcare, teleconference, telemedicine

## Abstract

Introduction: Japan has experienced a steady decline in live births and maternity facilities, with rural areas particularly affected by shortages of obstetricians and restricted access to perinatal care. Telemedicine has been proposed as a means of bridging these gaps, yet doctor-to-doctor systems remain scarcely reported, especially in obstetrics. This study aimed to evaluate the feasibility, clinical utility, and educational impact of a doctor-to-doctor teleconsultation system linking a tertiary university hospital with a rural municipal maternity facility in Japan.

Methods: We conducted a retrospective observational study of pregnant women whose management was discussed via a teleconsultation over a 17-month period (November 2023-March 2025). Clinical data and pregnancy outcomes were reviewed. Six junior physicians who used the system completed an anonymous questionnaire consisting of seven Likert-scale items and three open-ended questions.

Results: During the study period, 21 teleconsultations were held, covering 58 cases. The main clinical issues were obstetric complications (20, 34%), non-obstetric maternal conditions (20, 34%), fetal anomalies (8, 14%), and early pregnancy loss (4, 6.9%). Management planning was the most frequent reason for consultation (47, 81%). Of 47 cases in which management was discussed, 17 (36%) were referred to tertiary care, while all 30 cases (64%) managed locally resulted in uncomplicated deliveries at the rural hospital. In the survey, more than 80% of junior physicians (5 of 6) reported benefits in clinical decision-making, learning opportunities, and psychological reassurance.

Conclusion: This pilot study suggests that doctor-to-doctor teleconsultation is feasible and clinically valuable, while also offering educational and psychological support for junior physicians, thereby serving as a practical strategy for sustaining safe perinatal care in resource-limited rural settings.

## Introduction

The number of live births in Japan has been declining steadily over the past five decades, falling below 700,000 in 2024 [1]. In 2005, approximately 1.08 million births were recorded; thus, the birth rate has decreased by >25% over the past 20 years [1]. In parallel, the number of maternity facilities has also decreased sharply, from 3,991 in 1995 to 1,945 in 2021, representing a 50% reduction over 25 years [2]. This trend is particularly pronounced in rural areas, where pregnant women face logistical barriers in accessing timely, safe, and high-quality perinatal care.

Telemedicine has emerged as a potential strategy for addressing such challenges in regional healthcare delivery. During the COVID-19 pandemic, telemedicine services expanded rapidly [3-5], and some facilities even experimented with conducting antenatal checkups online in a doctor-to-patient format [6]. However, regulatory and operational constraints have hindered their widespread adoption, and many of these initiatives have been discontinued after the pandemic subsided, preventing permanent integration into routine care [7]. Against this background, doctor-to-doctor teleobstetric systems, which enable direct collaboration between physicians, remain scarce, both in Japan and globally, and their clinical utility and feasibility have not been thoroughly evaluated.

In Kyoto Prefecture, Japan, the Perinatal Medical Network initiative was launched to improve the quality of perinatal care and enhance the working conditions for obstetricians. This program connects the Prefectural Comprehensive Perinatal Medical Center with regional maternity facilities using information and communication technology (ICT), enabling timely case-specific clinical advice from tertiary centers. As the first stage of this program, in 2023, a teleconsultation partnership was established between the tertiary university hospital and the rural municipal hospital. The municipal hospital is the only maternity facility in the city that manages approximately 120 deliveries annually. However, the delivery team comprised one obstetrician aged >70 years and one junior obstetrician in their fourth or fifth postgraduate year who was dispatched from the university hospital. The nearest tertiary facilities are located 40 and 60 minutes away by car. Under these human and medical resource constraints, only low-risk cases can be managed locally, and high-risk cases must be identified in advance and appropriately referred or transferred. Failure to accurately triage such cases could severely compromise maternal and neonatal outcomes, as the limited resources at the rural municipal hospital make rapid responses to obstetric emergencies particularly challenging. Moreover, assigning junior physicians to work in rural hospitals without adequate support systems can exacerbate professional isolation, increase the psychological burden, and increase uncertainty in clinical decision-making.

This study aimed to evaluate the feasibility of a teleconsultation system designed to enable real-time collaboration between a tertiary urban center and a rural maternity facility under geographic and resource constraints. Specifically, we examined its operational implementation, clinical utility, and potential educational benefits, with particular attention paid to its role in enhancing clinical decision-making and supporting psychological safety among junior physicians.

## Materials and methods

This retrospective observational study was approved by the Institutional Review Boards of Kyoto University Hospital and Yasaka Hospital, a rural municipal hospital (approval numbers: R5062 and R07-005, respectively).

The TELEPRO (Tenma Shimon Co., Ltd., Tokyo, Japan) telemedicine system was utilized, which enabled simultaneous access from both hospitals, allowing bidirectional video communication. The system was also capable of the real-time sharing of ultrasound images, cardiotocography (CTG) tracings, electronic medical record screens, and live videos from a consultation room. Data sharing was limited to real-time transmission only, and the system did not allow the storage or sharing of recorded data.

The study included pregnant women attending Yasaka Hospital whose management plans were discussed via the teleconsultation system with Kyoto University Hospital between November 2023 and March 2025 (a 17-month period). Cases with insufficient clinical information regarding pregnancy course or delivery outcomes were excluded. If the same patient underwent multiple teleconsultations for distinct clinical issues, each session was considered an independent case for analysis.

During the same period, all six junior physicians who had worked at Yasaka Hospital and used the teleconsultation system were invited to complete a self-administered anonymous questionnaire (Table 1). These physicians were in their fourth or fifth postgraduate year, were undergoing specialty training in obstetrics and gynecology, and had not yet obtained board certification. The questionnaire, consisting of seven Likert-scale items and three open-ended questions, was designed to assess the physicians’ perceptions of the system’s usability, operational feasibility, and impact on clinical decision-making support.

**Table 1:** Questionnaire items

Likert-scale items (strongly agree, agree, neither agree nor disagree, disagree, or strongly disagree)
1. Did the telemedicine system assist you in making clinical decisions?
2. Did the presence of the telemedicine system provide you with a sense of psychological reassurance when managing challenging cases?
3. Was remote collaboration between physicians effective in improving the quality of care?
4. Do you feel that participating in teleconsultations enhanced your medical knowledge and clinical decision-making skills?
5. Was the system valuable as a learning opportunity for junior physicians?
6. Are you satisfied with the operability and ease of use of the system?
7. Do you believe that this system could be applicable to healthcare in other regions?
Open-ended items
8. Please describe the advantages of this system.
9. Please describe any difficulties or areas for improvement in this system.
10. Please describe any additional support you would expect for this system.

The questionnaire was independently developed by the authors to assess the usability, educational value, and psychological impact of the teleconsultation system, as no validated instruments addressing these aspects in obstetric doctor-to-doctor telemedicine were available. The draft questionnaire was reviewed by senior supervisors among the co-authors to ensure clarity, relevance, and that the items did not impose a psychological burden on respondents. Given the exploratory nature of this pilot study, formal psychometric validation was not performed. All questionnaire items were fully completed by all six respondents, and no missing or incomplete responses were identified. Free-text responses were reviewed descriptively. Similar responses were grouped together based on content, and all responses were summarized and presented in the "Results" section.

The primary outcomes were the number of teleconsultations, transfer rates to tertiary hospitals, and pregnancy outcomes. Secondary outcomes included the educational and psychological support provided to junior physicians. Patient data, including demographic characteristics (maternal age, gestational age, and obstetric history), pregnancy course, delivery outcomes, and transfer status, were retrospectively collected from anonymized medical records and analyzed using descriptive statistics. All descriptive analyses were performed using Microsoft Excel 2021 (Microsoft Corp., Redmond, WA). This study was reported in accordance with the STROBE (Strengthening the Reporting of Observational Studies in Epidemiology) guidelines.

## Results

Teleconsultation utilization and case characteristics

Senior obstetricians from the tertiary university hospital, each with >15 years of clinical experience, provided onsite clinical support to the rural municipal hospital on a rotating basis and spent eight days per month supervising and training junior physicians working there. During these onsite periods, in-person conferences were held with junior physicians to discuss complex obstetric cases. The teleconsultation system was primarily used for unscheduled conferences outside regularly scheduled in-person meetings.

Over the 17-month study period, the system was used 21 times, during which 58 cases were discussed via online teleconsultation. Each teleconsultation session typically involved the discussion of two to four cases. Of the 60 cases, two were excluded from the analysis because subsequent pregnancy course and delivery outcomes were unavailable, leaving 58 cases included in the final analysis. The median maternal age was 31 years (range, 19-41 years), with 20 (34%) nulliparous and 38 (66%) multiparous women. Twelve cases (21%) involved inpatients, whereas the majority of consultations were for outpatients (46 cases, 79%).

**Figure 1:**
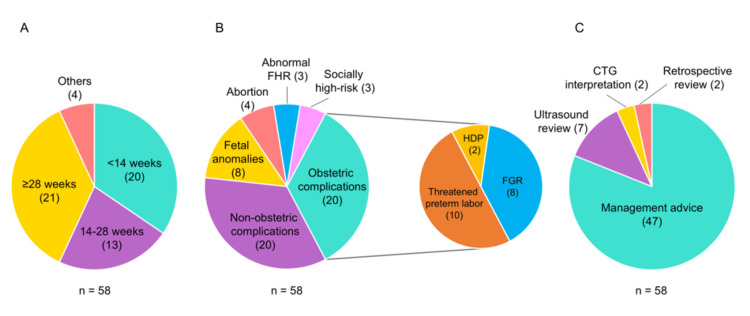
Clinical characteristics and reasons for consultation in telemedicine-supported obstetric cases (A) Distribution of gestational age at the time of consultation. (B) Main clinical issues or diagnoses. (C) Primary reasons for consultation. FHR: Fetal heart rate; HDP: Hypertensive disorders of pregnancy; FGR: Fetal growth restriction; CTG: Cardiotocography.

Regarding the gestational age at the time of consultation, 20 cases (34%) were in the first trimester (<14 weeks), 13 (22%) were in the second trimester (14-28 weeks), and 21 (36%) were in the third trimester (≥28 weeks) (Figure 1, Panel A). The main clinical issues or diagnoses were obstetric complications and non-obstetric maternal complications (e.g., obesity, asthma, and psychiatric disorders), each accounting for 20 cases (34%), as shown in Figure 1 (Panel B). The primary purpose of consultation was to seek advice on management plans in 47 cases (81%), as shown in Figure 1 (Panel C). Among the 47 cases in which management plans were discussed, 17 (36%) were referred to tertiary care facilities, whereas all 30 (64%) for which continued rural management was advised were managed and delivered at the Yasaka Hospital. In these 30 cases, the median gestational age at delivery was 39 weeks and 5 days (range: 37 weeks and 0 days to 40 weeks and 5 days), and the median birth weight was 3,186 g (range: 2,562-3,848 g). The median umbilical artery pH was 7.30 (range: 7.10-7.39). No maternal complications were observed. One neonate required postnatal transfer to a tertiary care facility because of respiratory distress.

Representative clinical cases

Case 1

**Figure 2:**
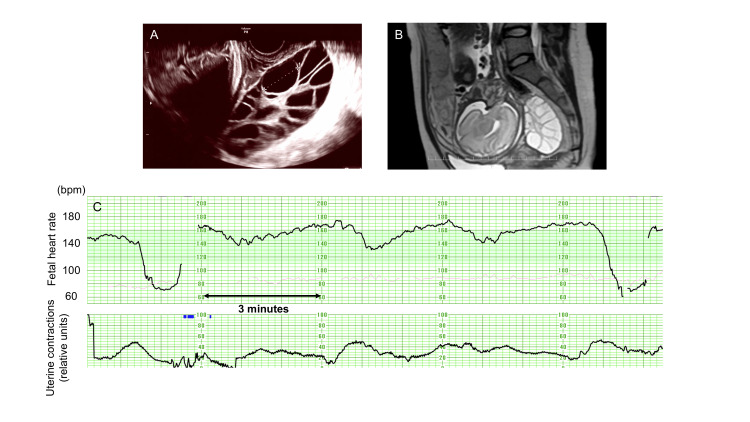
Representative clinical images from the telemedicine system (A, B) Enlarged ovary in the pouch of Douglas in case 1 is observed on transvaginal ultrasound (A) and T2-weighted sagittal magnetic resonance imaging (B). (C) Cardiotocography tracing from case 3 showing various deceleration patterns.

A 31-year-old nulliparous woman was found to have bilateral ovarian enlargement, measuring 100 × 53 mm in the right ovary and 102 × 99 mm in the left ovary, during a routine antenatal checkup at 30 weeks of gestation. Ultrasound images were shared via the teleconsultation system (Figure 2, Panel A). Obstetricians at the tertiary university hospital advised the junior obstetrician to perform serum human chorionic gonadotropin (hCG) measurements and pelvic magnetic resonance imaging (MRI). Based on these findings (hCG level of 35,400 mIU/mL and MRI), hyperreactio luteinalis was suspected rather than an ovarian tumor, and referral to a tertiary care center was recommended (Figure 2, Panel B). At 40 weeks of gestation, the patient delivered a 3,674 g male infant via cesarean delivery at the referral facility.

Case 2

A 29-year-old nulliparous woman was diagnosed with fetal growth restriction (FGR) at approximately 24 weeks of gestation, with an estimated fetal weight of approximately -2.0 standard deviation (SD). Because similar findings persisted at 26 weeks, a management plan was discussed using the teleconsultation system. Although no fetal structural abnormalities were detected on ultrasound examination performed by a junior physician, neonatal care was expected after delivery, and referral to a tertiary center was advised. The patient subsequently developed hypertensive disorders of pregnancy (HDP) at 31 weeks and delivered a male infant weighing 1,206 g (-3.3 SD) via emergency cesarean delivery at 34 weeks of gestation.

Case 3

A 31-year-old multiparous woman, who had previously delivered her first child vaginally at the rural municipal hospital, presented with lower abdominal pain at 37 weeks of gestation. The CTG showed repeated decelerations (Figure 2, Panel C). The attending obstetrician consulted the tertiary university hospital via the teleconsultation system and shared the CTG tracings in real time. Based on clinical symptoms and fetal heart rate patterns, abruptio placentae was strongly suspected. Given the time required for maternal transfer and the coincidental presence of an anesthesiologist at Yasaka Hospital on that day, the team at Kyoto University Hospital advised immediate cesarean delivery at Yasaka Hospital. Intraoperatively, partial placental separation and clotted blood were observed, confirming the diagnosis of abruptio placentae. A female infant weighing 2,562 g was delivered with Apgar scores of 9 and 10 and an umbilical artery pH of 7.22.

Survey results

**Figure 3:**
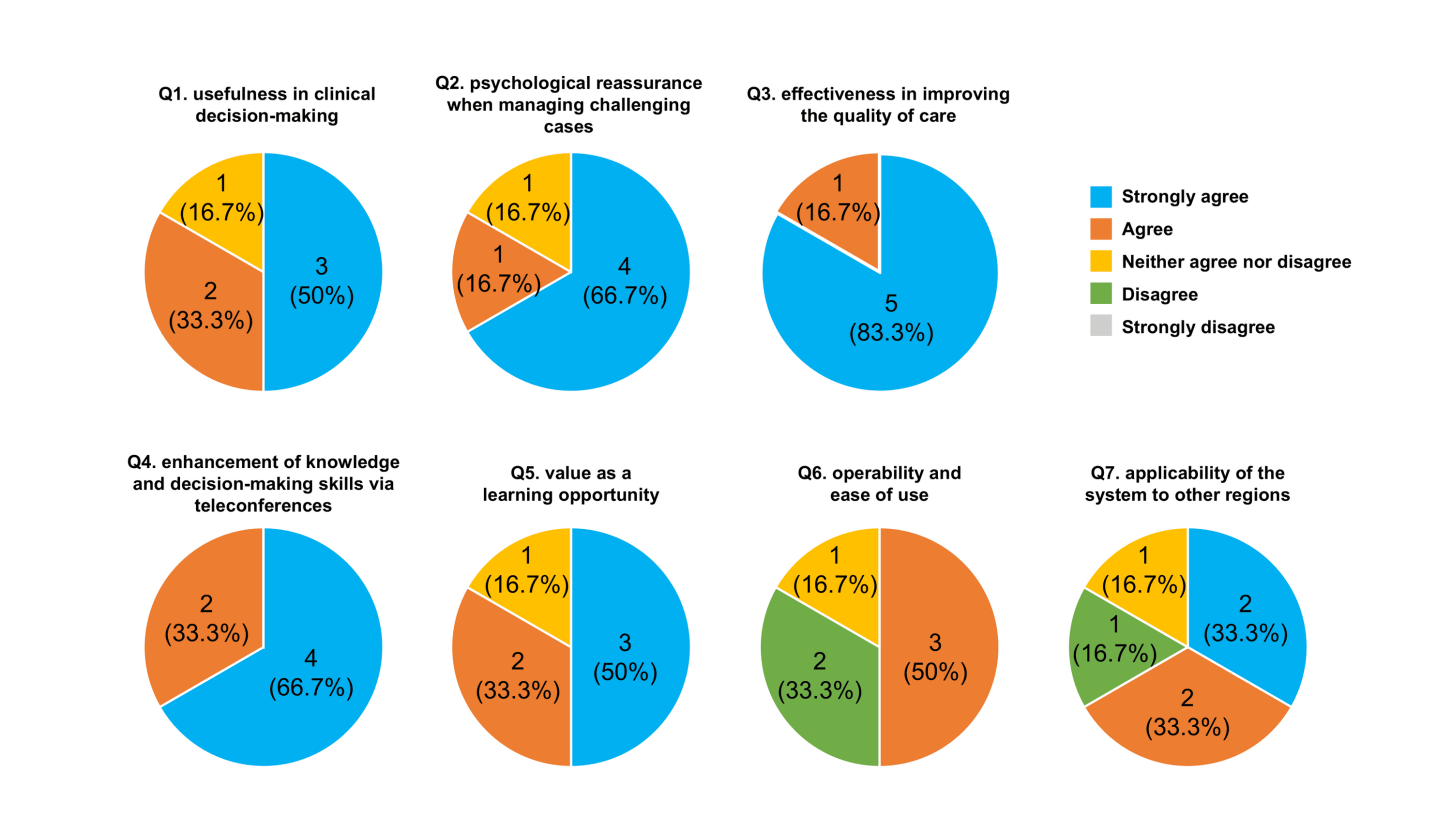
Results of the Likert-scale questionnaire administered to junior obstetricians regarding the teleconsultation system (n = 6) Q: Question.

The results of the questionnaire administered to junior obstetricians who had used the teleconsultation system are shown in Figure 3. During the study period, six junior physicians utilized this telemedicine system, and complete responses were obtained from all participants (n = 6). For Questions 1 (usefulness in clinical decision-making) and 5 (value as a learning opportunity), >80% of the respondents provided positive ratings. Similarly, for Question 2 (psychological reassurance when managing challenging cases), >80% of respondents responded “agree” or “strongly agree.” Furthermore, all respondents provided positive ratings for Questions 3 (effectiveness in improving the quality of care) and 4 (enhancement of clinical decision-making skills through participation in teleconsultation). In contrast, half of the respondents provided negative evaluations for Question 6 (operability and ease of use). For Question 7 (applicability of the system to other regions), approximately two-thirds of the respondents provided positive responses.

In the free-text section of Question 8 (advantages of the system), all respondents cited the ability to share clinical information in real time as a major benefit. The simultaneous sharing of medical records and images during case discussions enabled accurate information transfer to senior obstetricians and facilitated timely instruction.For Question 9 (areas for improvement), multiple respondents reported issues such as unstable connections; the system being installed only in outpatient consultation rooms, making it inconvenient for ward-based consultations; and the need for an asynchronous communication function, similar to email, to avoid coordinating schedules with supporting physicians. Finally, in response to Question 10 (expectations for the system), the participants suggested that the system should be more compact and portable, regular scheduling of teleconsultation should be institutionalized, and the system should be disseminated nationwide.

## Discussion

In Japan, where the decline in maternity facilities and shortage of obstetricians in rural areas have become increasingly severe, this study represents the first report demonstrating the operational implementation and clinical utility of a telemedicine system integrated into limited perinatal care resources. Our findings suggest that such a system may also contribute to providing educational support for junior obstetricians working in small teams in rural hospitals and enhance their psychological safety in clinical decision-making.

With advances in ICT, the feasibility of telemedicine has increased dramatically in recent years; however, its application in obstetric practice remains limited. Telemedicine is generally best suited for conditions with low urgency, where regular follow-up is central, and sufficient diagnostic information can be obtained through imaging and patient interviews alone. Indeed, telemedicine has gained widespread acceptance in fields that meet these criteria, such as diabetes management [8] and psychiatry [9,10]. In contrast, in obstetrics, attempts were made during the COVID-19 pandemic to conduct antenatal checkups remotely [6], and patient satisfaction was not necessarily low [11]. However, these efforts have failed to achieve widespread adoption [7]. A key reason lies in the unique structural challenge of obstetrics, in which both the mother and fetus must be simultaneously assessed. Maternal care can be partially managed through video consultations, blood pressure monitoring, and urinalysis; however, fetal assessment requires essential examinations, such as Doppler auscultation of the fetal heart and ultrasonographic evaluation of fetal growth, which can only be performed in person. Consequently, doctor-to-patient telemedicine in obstetrics faces inherent structural limitations, as fetal evaluations cannot be fully replaced by remote methods. Moreover, although pregnancy has a relatively short duration, maternal and fetal conditions change rapidly and dynamically, making routine follow-up-based telemedicine less applicable to obstetric care than to other medical fields.

In contrast, the present study demonstrated that doctor-to-doctor telemedicine can provide substantial benefits in facilities where even a small number of obstetricians are present. A randomized controlled trial conducted in the Netherlands compared home telemonitoring with the inpatient management of pregnancies complicated by HDP or FGR [12]. The study found that home telemonitoring was non-inferior to inpatient care with respect to the primary outcome, defined as a composite of adverse perinatal outcomes; however, regular visits and guidance from qualified healthcare providers were prerequisites [12]. This finding suggests that when a facility has a minimal number of physicians with adequate clinical competence, the addition of telemedicine support may enable the provision of high-quality obstetric care. Similar evidence has been reported in other high-acuity fields, such as trauma care [13] and military medicine [14], where telemedicine has improved access to specialized expertise in rural or frontline settings, enhanced detection of severe cases, and reduced unnecessary patient transfers. These findings support the present study, which sought to optimize the use of limited human resources while maintaining safety. In our study, all cases deemed manageable at the rural hospital through teleconsultation were ultimately delivered safely at Yasaka Hospital, underscoring the value of such collaborative discussions in accurately distinguishing between high- and low-risk cases.

One notable strength of this study was that it examined how a telemedicine system can benefit junior physicians working in regional hospitals. Doctor-to-doctor telemedicine has been categorized into four domains: diagnostic support, medical treatment support, information sharing, and education and learning [15]. However, empirical evaluations of its educational impact remain scarce. For example, in Canada, a multidisciplinary teleconsultation on interstitial lung disease that linked regional hospitals with a tertiary center in Toronto was reported to enhance junior physicians’ confidence in diagnosis and management [16]. This aligns with our finding that junior physicians perceived improvements in their knowledge and decision-making skills through participation in the teleconsultation system. Similarly, a study from Italy demonstrated that the more frequent use of teleconsultation improved relational coordination among physicians, thereby promoting accurate information sharing and effective collaboration in clinical decision-making [17]. Simultaneously, the sustainable implementation of doctor-to-doctor telemedicine requires dedicated personnel, and shortages in this regard are a major limiting factor [15]. In Japan, junior obstetricians must undergo training in regional hospitals as part of their specialist education, and senior physicians affiliated with academic medical centers are responsible for providing them with adequate educational and psychological support. Collaboration with government authorities will be essential to secure the financial resources that ensure the sustainability of such systems.

This study had several limitations. First, it was conducted at a single institution with a limited number of cases, restricting the generalizability of the findings to other regions. Second, although the questionnaire responses were collected anonymously, the respondents were physicians who directly benefited from the educational support of the system, raising the possibility of response bias. Third, the observation period was relatively short, and fully assessing long-term outcomes, such as improvements in maternal and neonatal prognosis, was difficult. Therefore, future research should include multicenter collaborations and long-term evaluations to further validate the effectiveness and sustainability of the system.

## Conclusions

This study demonstrated that a telemedicine system linking a tertiary urban center with a regional hospital is feasible and has clinical utility in the context of obstetric care under regional constraints. Moreover, the system was suggested to provide educational support and enhance psychological safety for junior physicians working in regional hospitals, thereby contributing to an improved quality of obstetric practice and potentially serving as a practical means of sustaining safe perinatal care in resource- and geography-limited settings.
